# Supplementary material for: Poly(N,N′-Diethylacrylamide)-Based Thermoresponsive Hydrogels with Double Network Structure
Source: Polymers (Basel). 2020 Oct 27;12(11):2502. doi: 10.3390/polym12112502 (PMC7692105; doi:10.3390/polym12112502)
Supplement: Supplementary file 1 [file polymers-12-02502-s001.zip › polymers-967934-supplementary.docx]

SUPPLEMENTARY MATERIALS

Poly(*N,N’*-diethylacrylamide)-based thermoresponsive hydrogels with double network structure

Lenka Hanyková^1,*^, Ivan Krakovský^1^, Eliška Šestáková^1^, Julie Šťastná^1^ and Jan Labuta^2^

^1^ Department of Macromolecular Physics, Faculty of Mathematics and Physics, Charles University, V Holešovičkách 2, 180 00 Prague 8, Czech Republic

^2^ International Center for Materials Nanoarchitectonics (WPI-MANA), National Institute for Materials Science (NIMS), 1-1 Namiki, Tsukuba, Ibaraki 305-0044, Japan

***** Correspondence: Lenka.Hanykova@mff.cuni.cz

**Figure S1.** (a) Temperature dependences of swelling ratio and (b) *p*-fraction for DNA1 hydrogel realized on two specimens (1^st^ and 2^nd^ experiment show good reproducibility).

**Figure S2.** Temperature dependence of swelling ratio for SN hydrogels. Experimental points are fitted according to Eq. (16).

**Figure S3.** Temperature dependence of swelling ratio for DNA hydrogels. Experimental points are fitted according to Eq. (16).

**Figure S4.** Temperature dependence of swelling ratio for DNM hydrogels. Experimental points are fitted according to Eq. (16).

**Figure S5.** DSC curves for SN hydrogels obtained during heating.

**Figure S6.** DSC curves for DNA hydrogels obtained during heating.

**Figure S7.** DSC curves for DNM hydrogels obtained during heating.

**
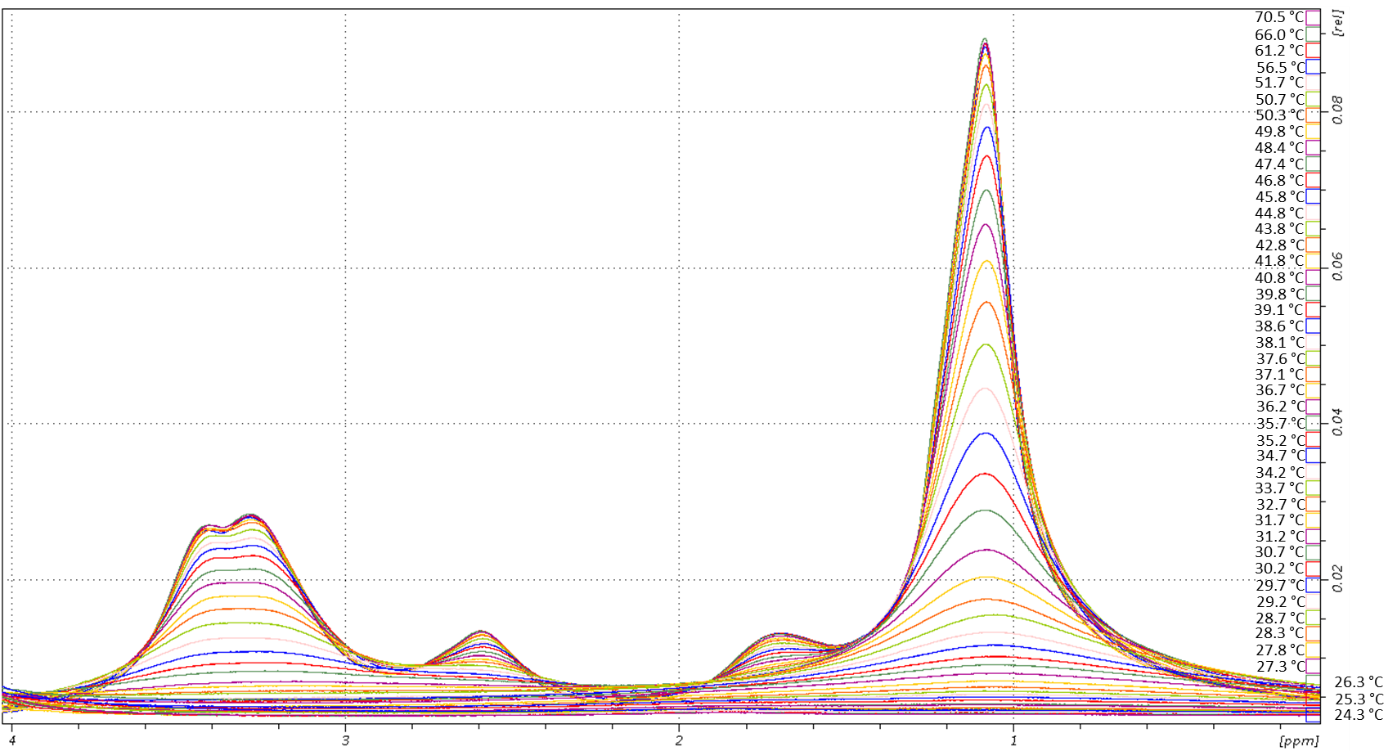
**

E

D

C

B

Increasing temperature

**Figure S8.** ^1^H spectra of hydrogel SN1 recorded at various temperatures. Peak assignments are explained in the text.


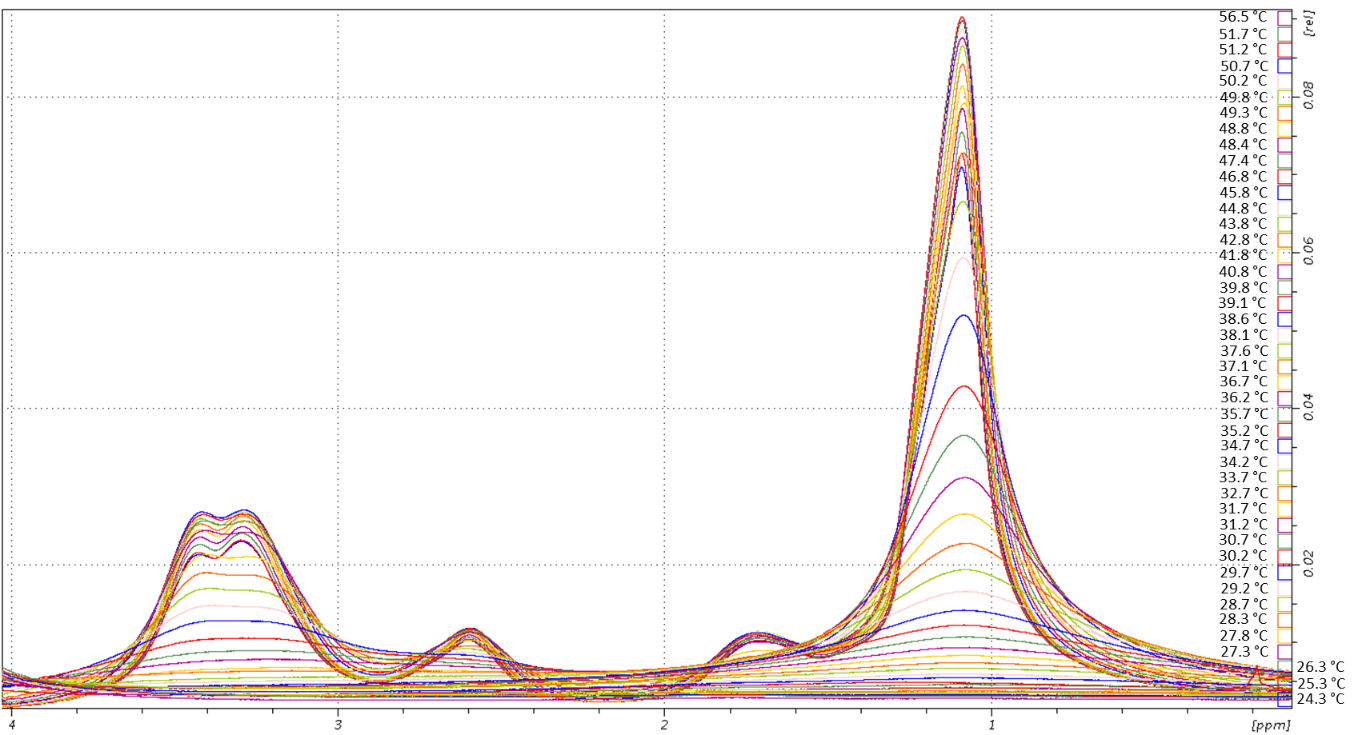


E

D

C

B

Increasing temperature

**Figure S9.** ^1^H spectra of hydrogel SN2 recorded at various temperatures. Peak assignments are explained in the text.


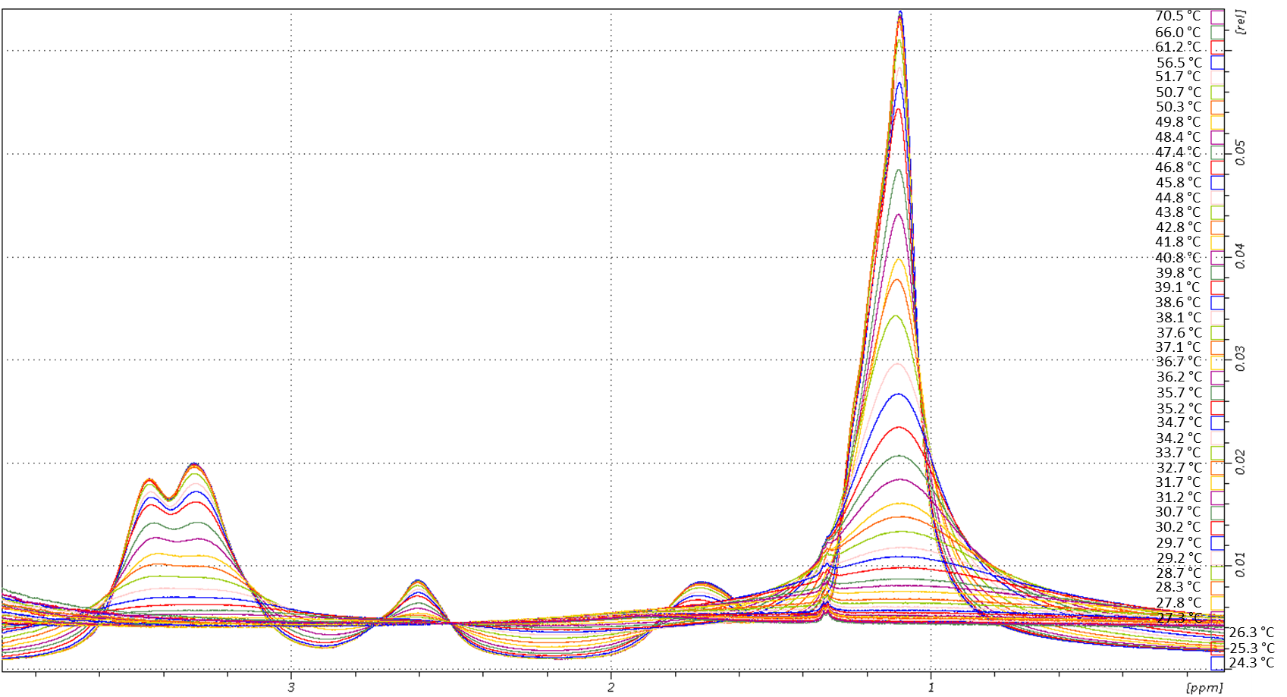


B

C

D

E

Increasing temperature

**Figure S10.** ^1^H spectra of hydrogel SN3 recorded at various temperatures. Peak assignments are explained in the text.


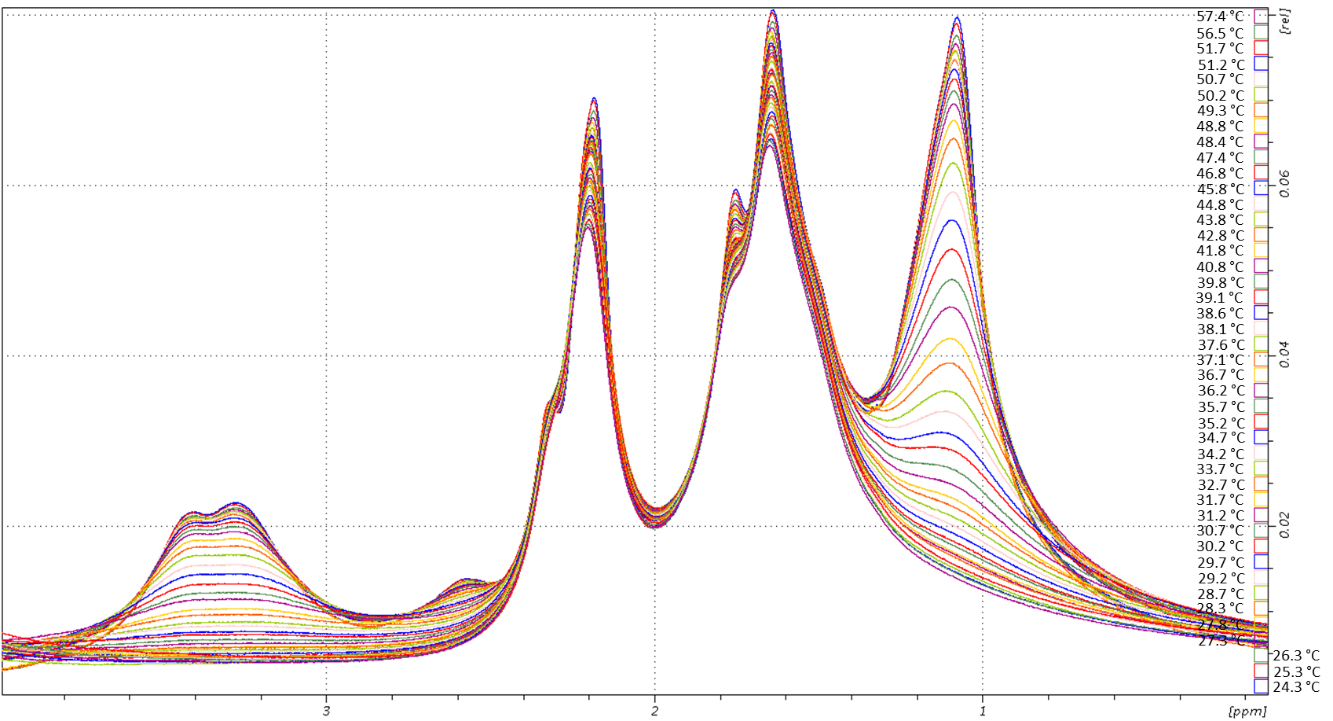


B

C

C’

D

E

Increasing temperature

**Figure S11.** ^1^H spectra of hydrogel DNA1 recorded at various temperatures. Peak assignments are explained in the text.


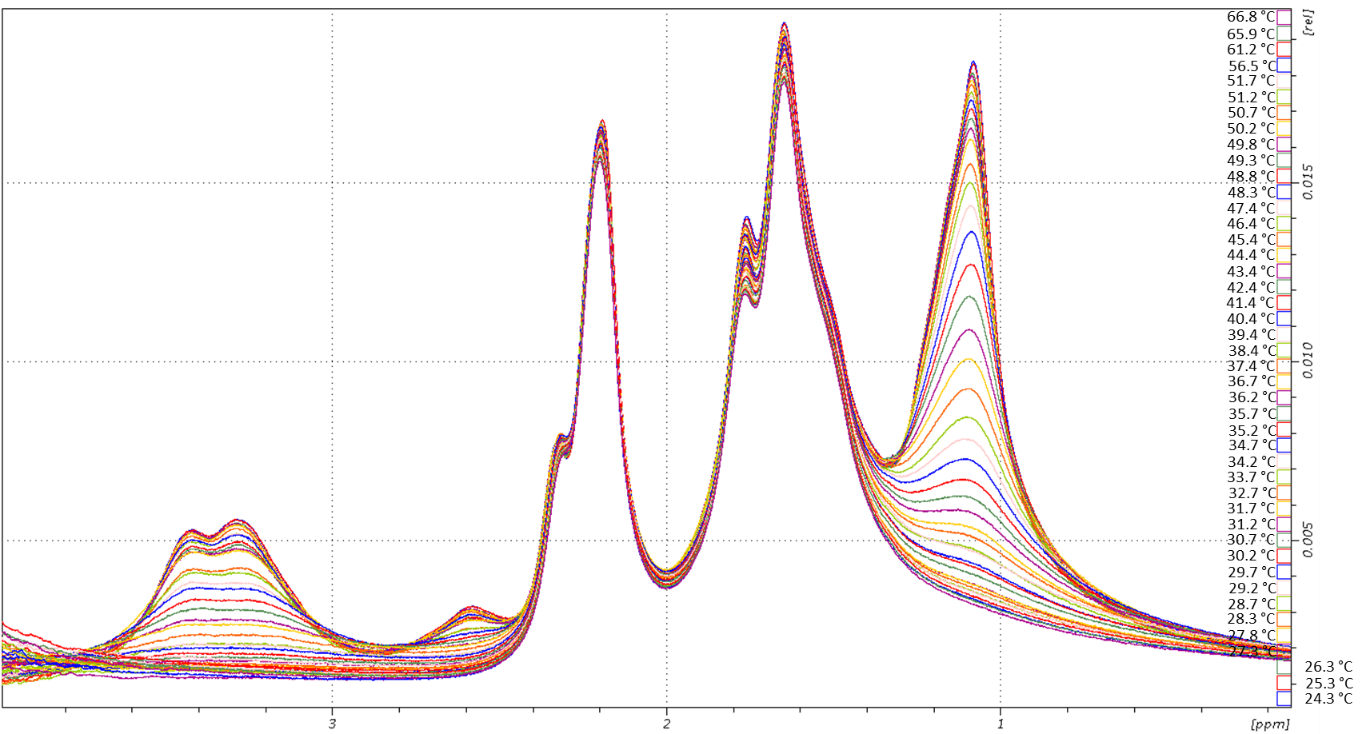


C’

D

E

Increasing temperature

C

B

**Figure S12.** ^1^H spectra of hydrogel DNA2 recorded at various temperatures. Peak assignments are explained in the text.


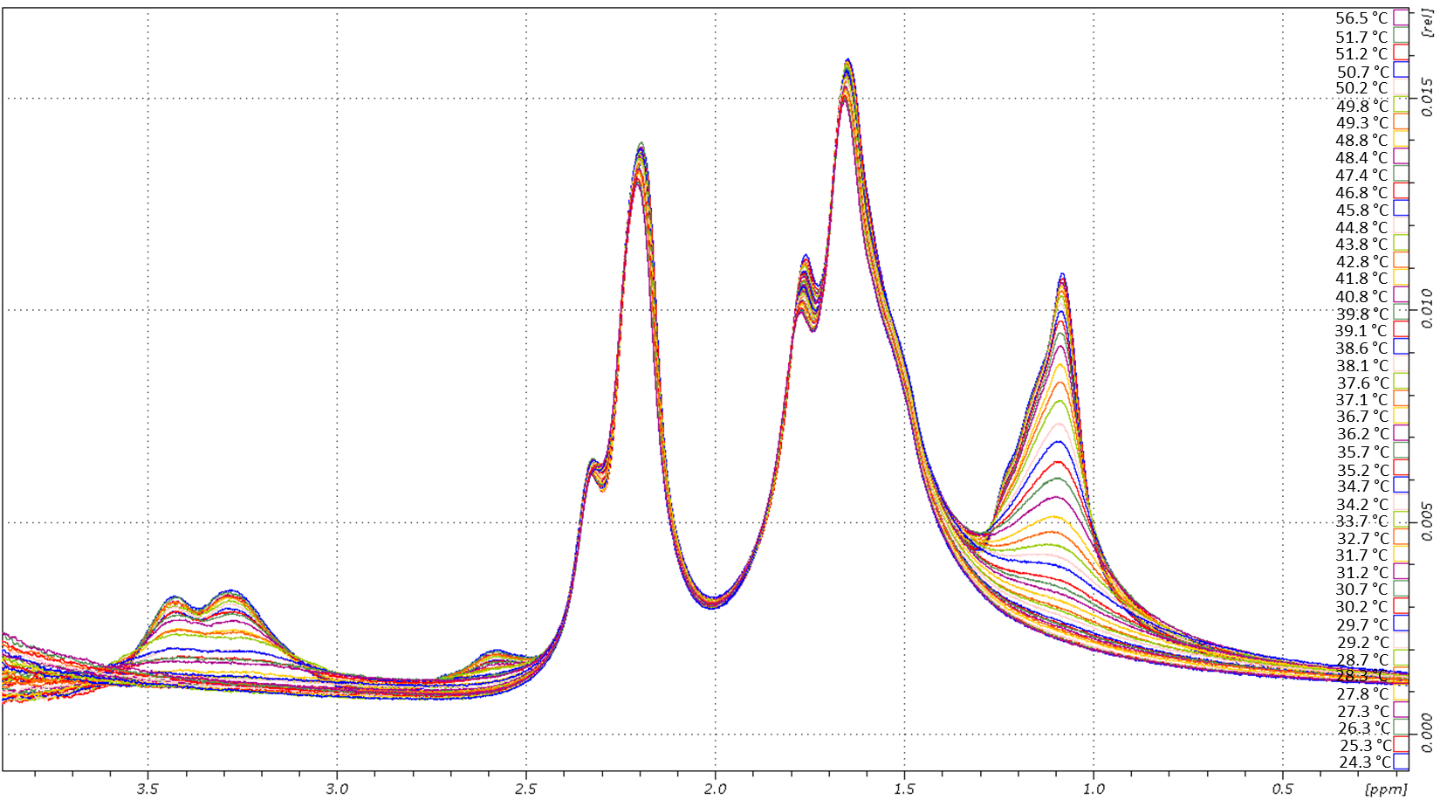


Increasing temperature

E

D

C’

C

B

**Figure S13.** ^1^H spectra of hydrogel DNA3 recorded at various temperatures. Peak assignments are explained in the text.


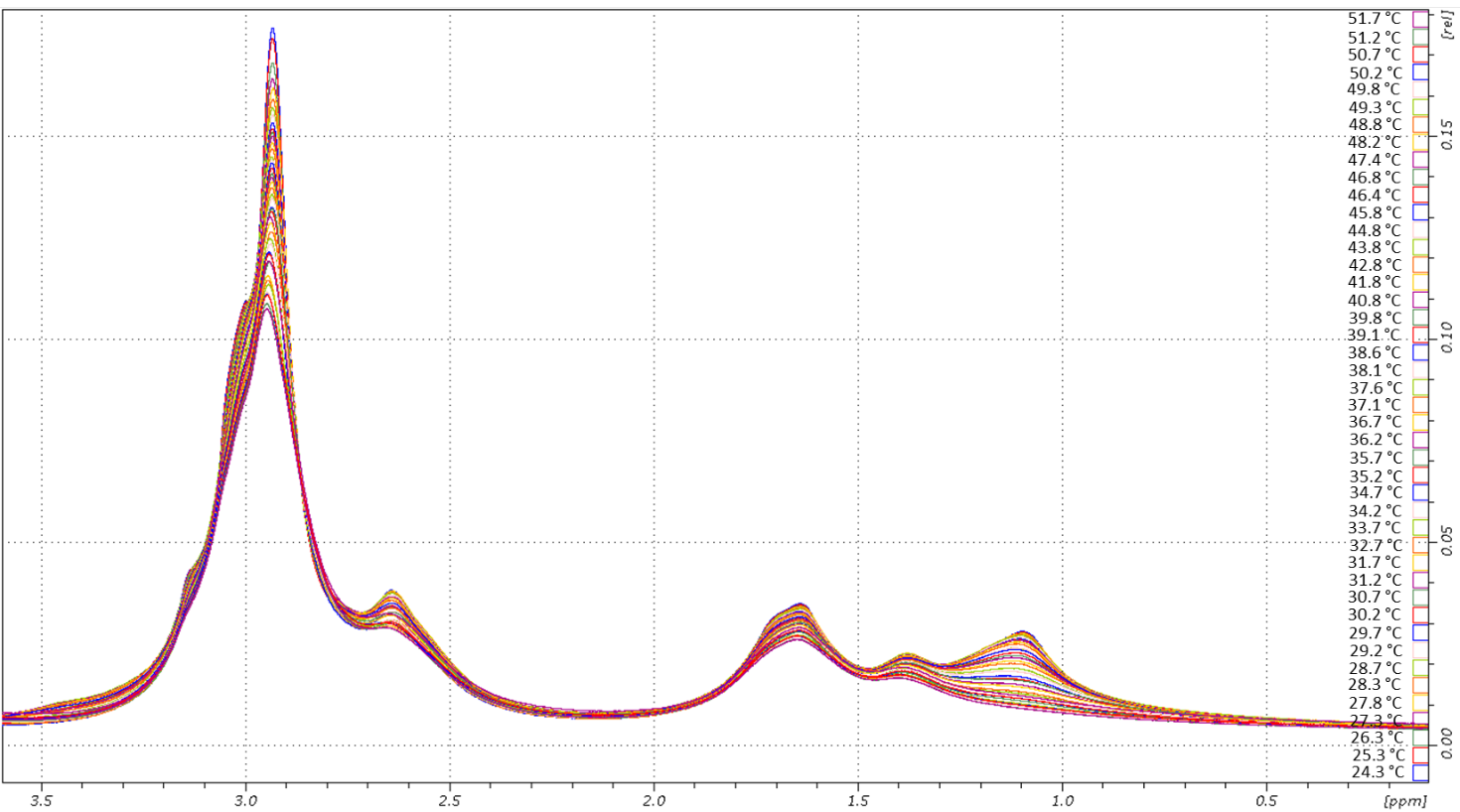


F

D’

B´

C

E

Increasing temperature

**Figure S14.** ^1^H spectra of hydrogel DNM1 recorded at various temperatures. Peak assignment: CH_3_ group of PDMAAm and CH_2_ group of PDEAAm (B’), CH main chain group of PDEAAm (C), main chain groups CH_2_ of PDEAAm and CH of PDMAAm (D’), main chain group CH_2_ of PDMAAm (F), CH_3_ group of PDEAAm (E).


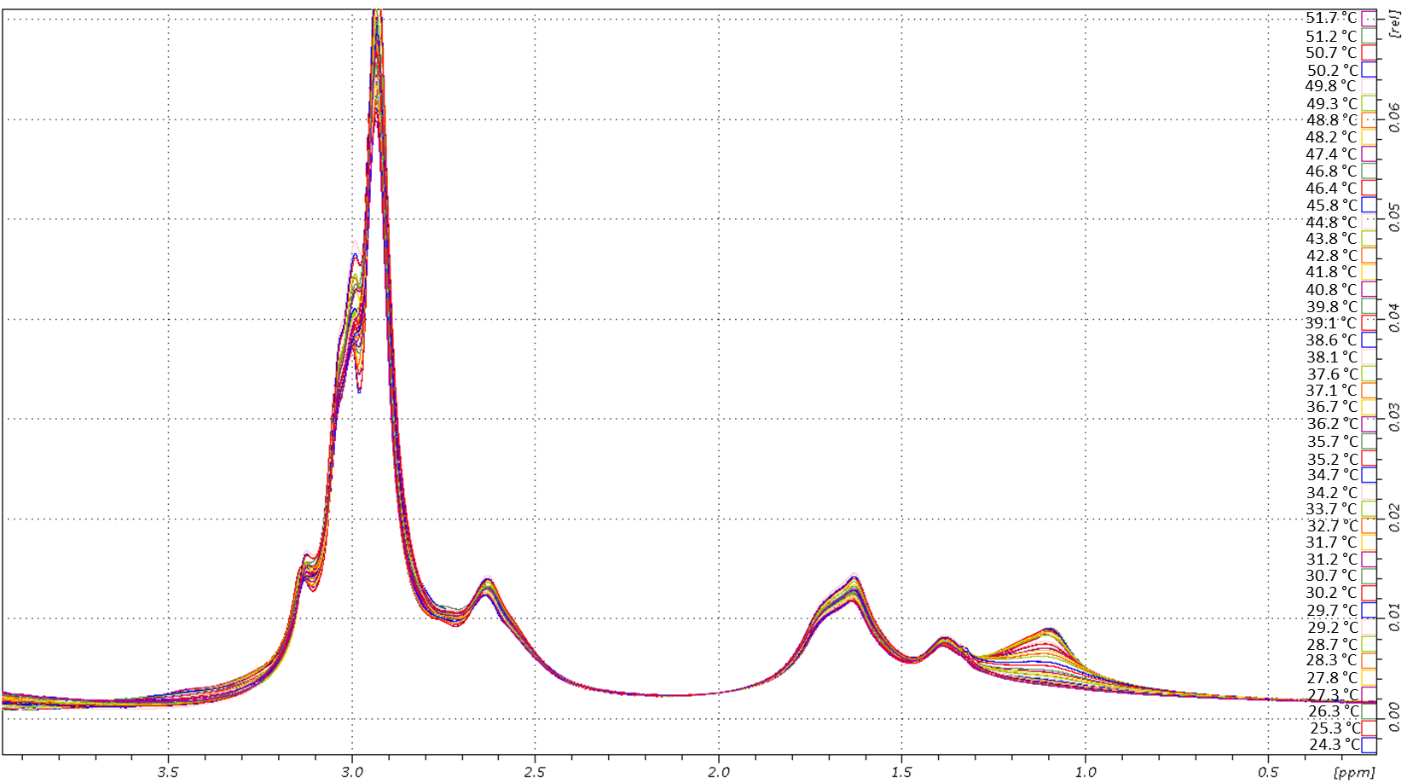


Increasing temperature

E

F

D’

C

B´

**Figure S15.** ^1^H spectra of hydrogel DNM2 recorded at various temperatures. Peak assignment: CH_3_ group of PDMAAm and CH_2_ group of PDEAAm (B’), CH main chain group of PDEAAm (C), main chain groups CH_2_ of PDEAAm and CH of PDMAAm (D’), main chain group CH_2_ of PDMAAm (F), CH_3_ group of PDEAAm (E).


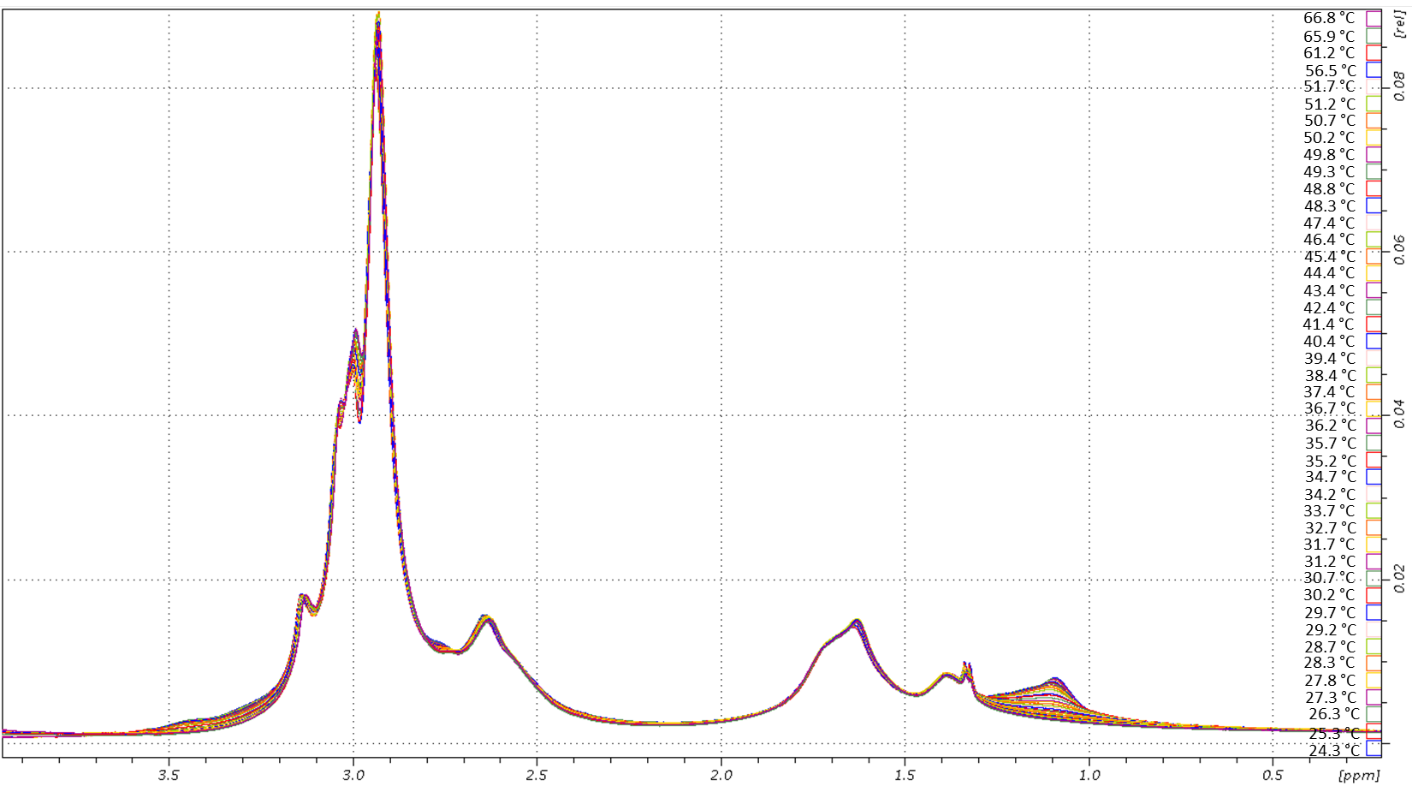


Increasing temperature

F

D’

B´

C

E

**Figure S16.** ^1^H spectra of hydrogel DNM3 recorded at various temperatures. Peak assignment: CH_3_ group of PDMAAm and CH_2_ group of PDEAAm (B’), CH main chain group of PDEAAm (C), main chain groups CH_2_ of PDEAAm and CH of PDMAAm (D’), main chain group CH_2_ of PDMAAm (F), CH_3_ group of PDEAAm (E).

**Figure S17.** Temperature dependences of *p*-fraction for DNM hydrogels. Experimental points are fitted according to Eq. (2).


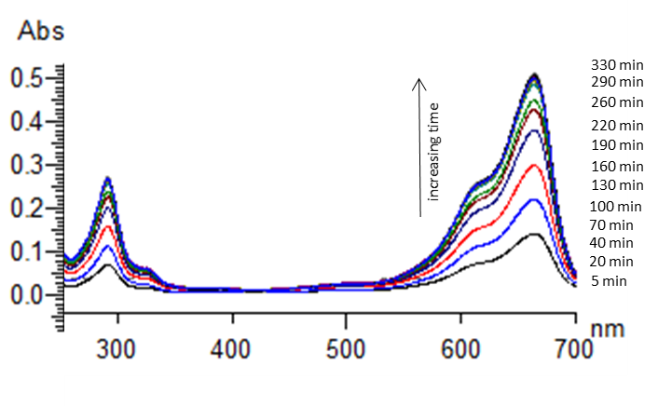


**Figure S18.** UV-Vis absorbance spectra of methylene blue released from hydrogel SN1 as a function of time.


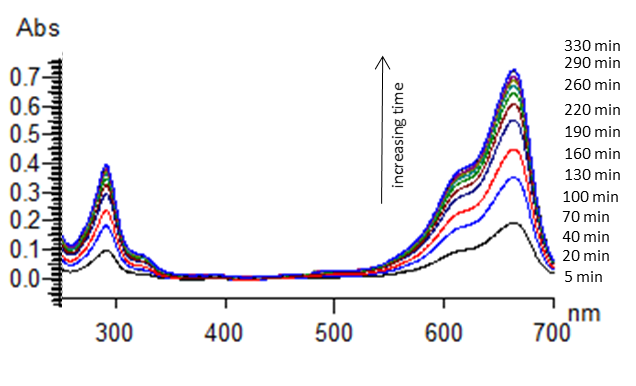


**Figure S19.** UV-Vis absorbance spectra of methylene blue released from hydrogel SN2 as a function of time.


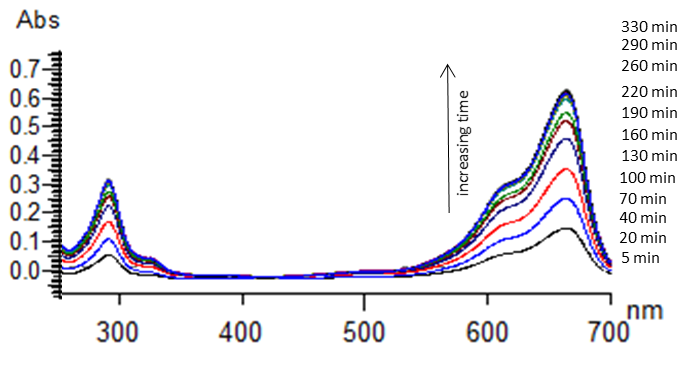


**Figure S20.** UV-Vis absorbance spectra of methylene blue released from hydrogel SN2 as a function of time.


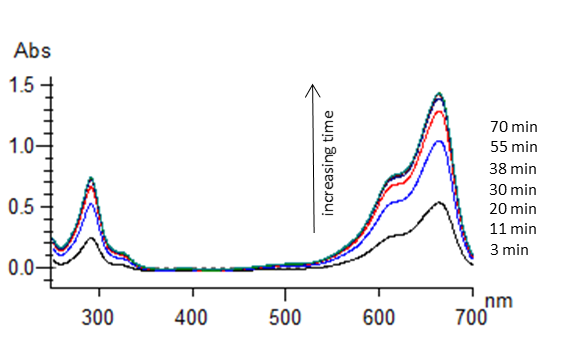


**Figure S21.** UV-Vis absorbance spectra of methylene blue released from hydrogel DNA1 as a function of time.


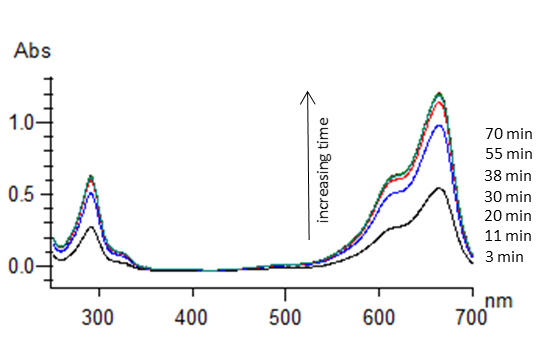


**Figure S22.** UV-Vis absorbance spectra of methylene blue released from hydrogel DNA2 as a function of time.


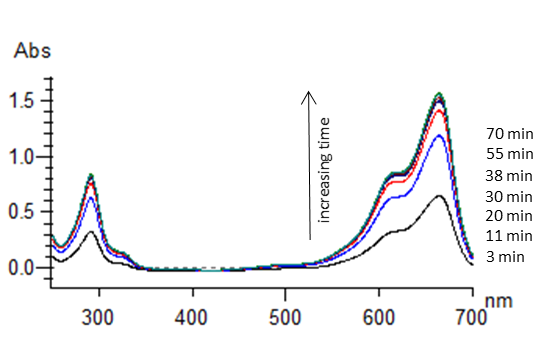


**Figure S23.** UV-Vis absorbance spectra of methylene blue released from hydrogel DNA3 as a function of time.


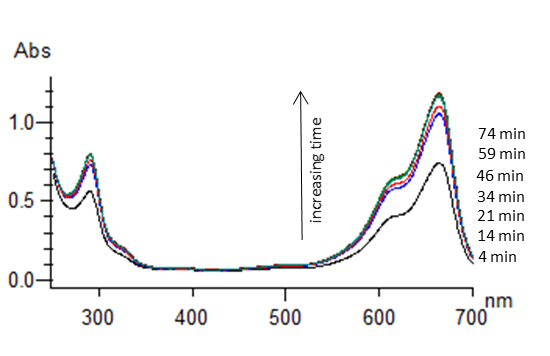


**Figure S24.** UV-Vis absorbance spectra of methylene blue released from hydrogel DNM1 as a function of time.


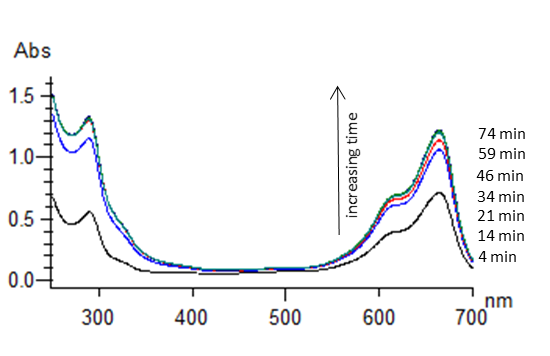


**Figure S25.** UV-Vis absorbance spectra of methylene blue released from hydrogel DNM2 as a function of time.


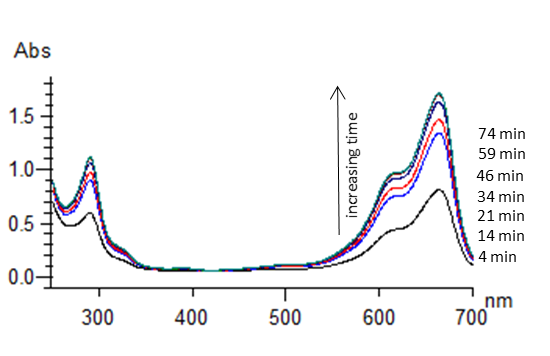


**Figure S26.** UV-Vis absorbance spectra of methylene blue released from hydrogel DNM3 as a function of time.

**Figure S27.** Time dependence of MB concentration for the release process for SN hydrogels. Experimental points are fitted according to Eq. (4).

**Figure S28.** Time dependence of MB concentration for the release process for DNA hydrogels. Experimental points are fitted according to Eq. (4).

**Figure S29.** Time dependence of MB concentration for the release process for DNM hydrogels. Experimental points are fitted according to Eq. (4).
